# STAT3 decoy oligonucleotide-carrying microbubbles with pulsed ultrasound for enhanced therapeutic effect in head and neck tumors

**DOI:** 10.1371/journal.pone.0242264

**Published:** 2020-11-18

**Authors:** Thiruganesh Ramasamy, Xucai Chen, Bin Qin, Daniel E. Johnson, Jennifer R. Grandis, Flordeliza S. Villanueva

**Affiliations:** 1 Center for Ultrasound Molecular Imaging and Therapeutics, Pittsburgh Heart, Lung, Blood and Vascular Medicine Institute, University of Pittsburgh, Pittsburgh, PA, United States of America; 2 Department of Otolaryngology-Head and Neck Surgery, University of California San Francisco, San Francisco, CA, United States of America; Columbia University, UNITED STATES

## Abstract

Signal transducer and activator of transcription-3 (STAT3) is an oncogenic transcription factor implicated in carcinogenesis, tumor progression, and drug resistance in head and neck squamous cell carcinoma (HNSCC). A decoy oligonucleotide targeting STAT3 offers a promising anti-tumor strategy, but achieving targeted tumor delivery of the decoy with systemic administration poses a significant challenge. We previously showed the potential for STAT3 decoy-loaded microbubbles, in conjunction with ultrasound targeted microbubble cavitation (UTMC), to decrease tumor growth in murine squamous cell carcinoma. As a next step towards clinical translation, we sought to determine the anti-tumor efficacy of our STAT3 decoy delivery platform against human HNSCC and the effect of higher STAT3 decoy microbubble loading on tumor cell inhibition. STAT3 decoy was loaded on cationic lipid microbubbles (STAT3-MB) or loaded on liposome-conjugated lipid microbubbles to form STAT3-loaded liposome-microbubble complexes (STAT3-LPX). UTMC treatment efficacy with these two formulations was evaluated *in vitro* using viability and apoptosis assays in CAL33 (human HNSCC) cells. Anti-cancer efficacy *in vivo* was performed in a CAL33 tumor murine xenograft model. UTMC with STAT3-MB caused significantly lower CAL33 cell viability compared to UTMC with STAT3-LPX (56.8±8.4% vs 84.5±8.8%, respectively, *p*<0.05). *In vivo*, UTMC with STAT3-MB had strong anti-tumor effects, with significantly less tumor burden and greater survival compared to that of UTMC with microbubbles loaded with a mutant control decoy and untreated control groups (*p*<0.05). UTMC with STAT3 decoy-loaded microbubbles significantly decreases human HNSSC tumor progression. These data set the stage for clinical translation of our microbubble platform as an imaged-guided, targeted delivery strategy for STAT3 decoy, or other nucleotide-based therapeutics, in human cancer treatment.

## Introduction

Head and neck squamous cell carcinoma (HNSCC) is the sixth most common cancer worldwide, with 49,670 cases in the United States and 600,000 cases globally in 2017 [1]. The high morbidity and mortality rates of HNSCC are generally attributed to late clinical presentation with advanced stage malignancy [2]. Despite advances in conventional therapies such as surgery, radiotherapy, and chemotherapy, the five-year survival rate of patients with HNSCC is ~40% and has improved only marginally over the past several decades [3–5]. Both tobacco-associated and HPV-linked HNSCC are associated with hyperactivation of signal transducer and activator of transcription-3 (STAT3) [6]. Numerous studies have reported that phosphorylation at a single tyrosine residue (Y705) in the transactivation domain of STAT3, by Janus associated kinase (JAK) or c-Src, results in the proliferation and maintenance of multiple cancers including HNSCC [7, 8] and correlates with lower survival rates [9]. Phosphorylated STAT3 dimerizes and transfers signals from the cytoplasm to the nucleus where it induces a series of genes including Bcl-2/Bcl-xL, cyclin D1, and c-Myc, among other STAT3 target genes. Upregulation of STAT3 has been associated with the therapeutic resistance of oncogenic pathway inhibitors in oncogene-driven models (EGFR, HER2, ALK, and MET) where targeting STAT3 reverses the drug resistant phenotype [10]. Therefore, STAT3 is considered an important therapeutic target for several reasons, including its role in suppressing cancer cell apoptosis and in multiple oncogenic pathways, such that its inhibition would effectively block the action of several upstream tyrosine kinases [11].

One approach to blocking the activity of STAT3 employs a decoy oligonucleotide, which is a 15-mer double-stranded oligonucleotide that binds to activated STAT3 dimers and competitively inhibits the binding of these dimers to target gene promoters [12, 13]. While a systemically stable cyclic STAT3 decoy has been shown to have an anti-cancer effect after intravenous injection, the opportunity to increase concentrations of the STAT3 decoy to the tumor region using targeted delivery approaches remains relatively unexplored [14]. Further, oligonucleotides in general have poor biomembrane permeability and are susceptible to nuclease degradation, which may necessitate repeat administration, hindering clinical translation [15, 16].

Microbubbles (1–5 μm in diameter), consisting of a gas-filled core that is stabilized by either lipid, surfactant, protein, or a polymer-based shell, could be an attractive drug delivery system [17]. Intravenously injected microbubbles expand and contract, or cavitate, under the influence of ultrasound, causing bioeffects which can promote the delivery of cell-impermeant oligonucleotides exclusively to sites exposed to the ultrasound beam. The combination of ultrasound and microbubbles, hereafter referred to as ultrasound-targeted microbubble cavitation (UTMC), ultimately facilitates internalization of encapsulated gene therapeutics at the ultrasound-exposed site [18] via incompletely understood mechanisms mediated by cavitation-induced shear stress. UTMC-mediated delivery of various nucleic acid therapies has been shown to increase their anti-cancer efficacy in cancers such as glioma, prostate, melanoma, pharyngeal, and pancreatic cancers [19–22]. We previously demonstrated that UTMC treatment with STAT3 decoy-loaded cationic lipid microbubbles inhibits the growth of murine skin squamous cell carcinoma xenografts [23]. However, the efficacy of our platform against human cancers is unknown, and maximal dosing of STAT3 decoy was limited by the loading capacity of the lipid cationic microbubbles used in our prior study.

Accordingly, to further promote clinical translation, we tested the hypothesis that UTMC-mediated delivery of STAT3 decoy to human HNSCC would inhibit tumor growth. Furthermore, we sought to determine if a microbubble carrying a higher amount of STAT3 decoy than previously tested by our group, by using decoy-loaded liposomes conjugated to microbubbles, would confer greater inhibition of human HNSCC xenograft tumors. We used human HNSCC cells (CAL33) *in vitro* to compare the efficacies of two microbubble formulations combined with ultrasound. Thereafter we tested the superior performing microbubble formulation to deliver STAT3 via UTMC in nude mice bearing CAL33 xenograft tumors.

## Materials and methods

### STAT3 decoy oligonucleotides

The double-stranded STAT3 decoy oligonucleotide and its mutant version were purchased from IDT Technologies (Coralville, IA, USA). Prior to loading onto microbubbles, oligonucleotides were ligated using T4 ligase (New England Biolabs, Ipswich, MA, USA) to form cyclic decoy [24]. The complete sequence of STAT3 decoy is 5´-GTAAATC-18-GATTTACGGGAAATG-18-CATTTCCC-3´. A mutant decoy was used as a control and differed from the STAT3 decoy by a single nucleotide base pair with the sequence pattern: 5´-TTAAATC-18-GATTTAAGGGAAATG-18-CATTTCCC-3´.

### Formulation of decoy-loaded cationic lipid microbubbles

We have previously reported the synthesis of STAT3 decoy-loaded cationic lipid microbubbles [23]. 1,2-distearoyl-*sn*-glycero-3-phosphocholine (DSPC), 1,2-distearoyl-*sn*-glycero-3-ethylphosphocholine (Chloride salt) (DSEPC), and 1,2-distearoyl-sn-glycero-3-phosphoglycerol, sodium salt (DSPG) were purchased from Avanti Polar Lipids (AL, USA). Polyethylene glycol-40 (PEG-40) stearate was purchased from Sigma-Aldrich (MO, USA). All other chemicals were of reagent grade and used according to the manufacturer’s guidelines. Stock solutions of multilamellar vesicles (MLV) were prepared by dissolving DSPC, DSEPC, DSPG, and PEG-40 stearate at a molar ratio of 100:43:1:4.5 in chloroform and dried using argon gas. The dried lipid film was hydrated using isotonic saline and sonicated using a bath Ultrasonic Cleaner (VWR, Model 75D, New York, USA) until all the lipids were evenly dispersed. To prepare decoy-loaded microbubbles, 200 μL of 4X lipid stock solution was diluted with saline to 800 μL in a glass vial using saline, and 10 μg of STAT3 decoy or mutant decoy was added and sealed. The headspace of the vial was filled with perfluorobutane gas (FluoroMed, LP, Round Rock, TX, USA) and vortexed for 45 s using a dental amalgamator, resulting in microbubbles carrying STAT3 decoy (STAT3-MB) or mutant decoy (STAT3-MB-mut) via charge-charge interaction. The loaded microbubbles were washed three times using centrifugation at 53 g for 5 min with saline and stored at 4–8°C until further use. The maximal loading of STAT3 decoy was determined by a previously described iterative process of optimization [23]. The amount of decoy bound to microbubbles was measured using agarose gel electrophoresis with Gel-Red (Biotium, Fremont, CA) staining.

### Liposome-conjugated microbubble formulation

#### Biotinylated lipid microbubble preparation

Lipid microbubbles were prepared from DSPC, 1,2-distearoylsn-glycero-3-phosphoethanol-amine-N-[biotinyl(polyethyleneglycol)-2000 (DSPE-PEG2000-Biotin) (Laysan Bio Inc, Arab, Alabama), and PEG-40 stearate as previously described [25]. Briefly, DSPC, DSPE-PEG2000-biotin, and PEG-40 stearate (2/1/1, w/w/w) were dissolved in chloroform. The chloroform was evaporated by flushing with argon, followed by overnight vacuum drying. The dried lipid film was rehydrated in saline (final lipid concentration as 10 mg/mL) for 1 h at room temperature. After a brief sonication using a bath Ultrasonic Cleaner (VWR, Model 75D) to dissolve any lipid debris, the lipid dispersion was sonicated with a 20 kHz probe (Heat Systems, Ultrasonics XL-2020, Newtown, CT, USA) in the presence of perfluorobutane gas (FluoroMed, LP). After sonication, the microbubbles were washed with 25 mL saline twice (floatation for 1 h) to remove any free lipid and were suspended in saline saturated with perfluorobutane. The resulting biotinylated lipid microbubbles were aliquoted in vials with perfluorobutane-filled headspace and stored at 4°C until use.

#### Biotinylated cationic liposome preparation

1,2-dioleoyl-3-trimethylammonium-propane (DOTAP), 1,2-dioleoyl-sn-glycero-3-phosphoethanolamine (DOPE) (both purchased from Avanti Polar Lipids, USA), and DSPE-PEG2000-biotin were individually dissolved in chloroform and mixed together with a molar ratio of 49:49:2. The chloroform was evaporated by flushing with argon and followed by overnight vacuum drying. The dried lipid film was rehydrated in phosphate buffered saline (PBS) for 30 min at 65°C. The crude liposomes were extruded through two stacks of 400 nm polycarbonate membrane 21 times at 65°C using a mini extruder (Avanti Polar Lipids, AL, USA). The free lipids were removed by passing through a Sephadex G-50 column (GE Health Care, Little Chalfont, UK) and the resulting biotinylated cationic liposomes were stored at 4°C until use.

#### Conjugation of cationic liposome to lipid microbubbles

The biotinylated cationic liposomes were conjugated to biotinylated lipid microbubbles via biotin-streptavidin interaction. The biotinylated microbubble suspension (1×10^9^ microbubbles in 5 mL of saline) was incubated with 4.15 mg of streptavidin (Thermo Fisher Scientific, Rockford, USA) for 60 min. The microbubbles were then washed twice with 1 mL of PBS by centrifugation at 200 g for 3 min to remove any free streptavidin. The resulting streptavidin-microbubble suspension was slowly added to saline containing excess amounts of the biotinylated cationic liposomes. After 60 min of incubation with constant agitation, the microbubbles were washed twice with 1 mL saline by centrifugation at 200 g for 3 min to remove unbound liposomes.

#### STAT3 decoy loading of liposome-microbubble complex

The liposome-microbubble complexes (lipoplex, or LPX) were incubated with differing amounts of STAT3 decoy or mutant decoy (2 μg to 20 μg in a tube containing 1×10^8^ microbubbles) for 20 min to allow decoy loading by electrostatic interaction. The resultant microbubble complexes carrying liposomes loaded with STAT3 decoy (STAT3-LPX) or mutant decoy (STAT3-LPX-mut) were washed twice with 1 mL saline by centrifugation at 200 g for 3 min. For quantification of STAT3 decoy on LPX, STAT3 decoy-loaded LPX complexes were centrifuged at 200g for 3 min to separate LPX from unbound STAT3 decoy. Both subnatant (unbound decoy) and supernatant (containing LPX) were harvested separately. The decoy bound to LPX complexes was released by incubation with heparin for 10 min and analyzed by agarose gel electrophoresis with Gel-Red staining. Using this method, it was found that saturation of the LPX with STAT3 decoy occurred at 5 μg in the tube, equivalent to 50 μg in 1×10^9^ microbubbles (data not shown).

### Particle size and charge characterization

The concentration and size distribution of microbubbles were determined using Coulter Multisizer 3 (Beckman Coulter, Miami, FL, USA). A sample of 5 μL was diluted in 10 mL of Isoton (Beckman Coulter, Fullerton, CA, USA) and measured in triplicate. The surface charge of the particles was measured using Zetasizer Nano ZS (Malvern Instruments, Westborough, MA, USA).

### Anti-tumor efficacy of STAT3 via lipofectamine complex

Lipofectamine complexes were formulated by mixing the appropriate proportions of STAT3 or mutant decoy and lipofectamine 2000 (Life Technologies, Waltham, MA, USA), and incubated for 5 min prior to the *in vitro* studies. The cultured CAL33 cells (see below) were treated with fresh media containing varying molar concentrations (25–200 nM) of lipofectamine-complexed STAT3 decoy or mutant decoy and further incubated for 48 h. Cell viability was evaluated by MTT assay (see below).

### Anti-tumor efficacy of UTMC-mediated STAT3 decoy delivery to human HNSCC *in vitro*

#### Cells

Human tongue squamous cell carcinoma CAL33 cells were purchased from ATCC (Manassas, VA, USA) and were cultured in high glucose DMEM media supplemented with 10% FBS and 1% antibiotic mixture. Additionally, CAL33-STAT-Luc cells expressing the luciferase for STAT3-responsive promoter were cultured. The cells were maintained at 5% CO_2_ and 37°C ambient conditions.

#### Ultrasound treatment protocol

CAL33 cells were seeded in a 12-well plate (seeding density 2×10^5^ per well) and allowed to grow for 24 h. Microbubbles were added to the culture media to achieve a final ratio of 10 microbubbles/cell. The wells were covered with media containing 2% FBS, sealed with polycarbonate membrane and inverted. Ultrasound was delivered with a single-element immersion transducer (A302S, 25.4 mm in diameter, Olympus NDT, Center Valley, PA, USA), driven by an arbitrary function generator (AFG3252, Tektronix, Beavertona, OR, USA) connected to a gated radio frequency power amplifier (250A250AM8, Amplifier Research, Souderton, PA, USA). The ultrasound field was calibrated with a 200-μm capsule hydrophone (HGL-0200, Onda Corp, Sunnyvale, CA, USA). Ultrasound pulses were delivered at 1 MHz, 0.50 MPa peak negative pressure, 10-μs pulse duration, and pulse interval 1 ms (duty cycle 1%), for a total of 10 s. The media was removed after UTMC and cells were replenished with fresh media (10% FBS and 1% antibiotic mixture) and incubated for 48 h. Cell viability and apoptosis assays and Western blot analyses were then performed (see below).

#### Cell viability and determination of STAT3 inhibition *in vitro*

Cells were seeded in 12-well plates (seeding density 2×10^5^ cells per well) and incubated for 24 h. STAT3-MB or STAT3-LPX, or their corresponding mutant-loaded formulations (STAT3-MB-mut or STAT3-LPX-mut), were added to the cells. The plates were then subjected to the ultrasound protocol described above, then incubated for 20 minutes; media was then removed and replaced with fresh media, followed by incubation for an additional 48 h.

The subsequent viability of CAL33 cells was evaluated by MTT assay as follows: Cells were treated with 200 μL of MTT reagent (1.25 mg/mL) per well and incubated for 4 h, followed by the addition of 200 μL of DMSO to dissolve the formazan crystals. Absorbance was measured at 570 nm using a microplate reader (Beckman plate reader, Brea, CA, USA). Cell viability was calculated by normalizing to that of untreated control cells.

Knockdown of STAT3 signaling was evaluated using CAL33-STAT-Luc cells expressing luciferase gene driven by a STAT3-responsive promoter. The cells were cultured in 12-well plates, exposed to loaded microbubbles, and subjected to the UTMC protocol as described above. Luciferase activity was measured with a luciferase assay kit (Promega, Madison, WI, USA) according to manufacturer’s guidelines and normalized to total protein as measured by Bradford assay (Thermo Fisher Scientific, Waltham, MA, USA).

#### Apoptosis assay *in vitro*

A quantitative apoptosis assay was performed by flow cytometry using Annexin V-FITC and propidium iodide (PI) staining (BioLegend Kit, San Diego, CA, USA) of the CAL33 cells (see detailed methods in the S1 File).

#### Western blot analysis

Target gene expression changes resulting from the various STAT3 decoy formulations were evaluated using Western Blot. CAL33 cells treated with UTMC (see above) were incubated for 48 h, washed twice with PBS, detached in the presence of scraping buffer, centrifuged, and lysed using lysis buffer. The extracted proteins were separated using SDS-PAGE gel and transferred to PVDF membrane (Millipore, MA, USA). The membrane was incubated with antibodies against Bcl-xL, cyclin D1, and GAPDH (see detailed methods in the S1 File).

### Antitumor efficacy of UTMC-mediated STAT3 decoy delivery to human HNSCC *in vivo*

#### Animals

Balb/c nude mice (Charles Rivers, Pittsburgh, PA, USA) bearing human xenograft HNSCC tumors were used to determine the growth inhibitory effect of UTMC-mediated STAT3 decoy delivery. All animal experiments were approved by the University of Pittsburgh Institutional Animal Care and Use Committee. All animal welfare considerations were followed as per guidelines of the University of Pittsburgh Institutional Animal Care and Use Committee. Anesthesia was provided using 2% inhaled isoflurane. The total duration of each experiment was 15 days and a total of 22 mice were used for the study. The mouse was euthanized on the last day of the experimental protocol (Day 15), or earlier if the tumor was observed to be beyond 1000 mm^3^, using anesthesia (5% of isoflurane for 5 min) [23]. In the no treatment control group, 3 of the 8 mice were euthanized before Day 15; in the STAT3-MB-mut + UTMC group, 4 of the 7 mice were euthanized on Day 12; in the STAT3-MB + UTMC group, all of the 7 mice survived to Day 15. The microbubble formulations (STAT3-MB or STAT3-MB-mut) described above were utilized for the *in vivo* studies described below.

#### Tumor model and experimental protocol

Mice were anesthetized and 1×10^6^ CAL33 cells were subcutaneously injected into the right flank. The tumors were allowed to grow to a volume of 80–100 mm^3^ prior to initiation of treatment.

On the first day of treatment (Day 0), mice were anesthetized and a chronic indwelling catheter was placed in the internal jugular vein for microbubble infusion as described previously [23]. UTMC treatment was performed on Days 0, 3, and 6 as follows: Microbubbles (500 μL suspension containing 1×10^9^ MBs formulated with 10 μg of STAT3 decoy) were infused into the jugular vein over 20 min via an infusion pump. Therapeutic ultrasound was delivered using the same single-element immersion transducer system described above for the *in vitro* studies. The therapy transducer was oriented directly over the tumor, tilted slightly toward the tail of the animal to avoid ultrasound delivery to the abdomen. Over a period of 25 minutes during microbubble infusion, UTMC was delivered at 1 MHz transmit frequency, 0.70 MPa peak negative pressure, 100-μs pulse duration, repeated 5 times every 1 ms, followed by a 2-sec waiting period to allow microbubble reperfusion into the treatment area (overall duty cycle 0.025%). The successful destruction and replenishment of microbubbles in the treatment area was confirmed by simultaneous contrast-specific ultrasound perfusion imaging with a clinical ultrasound imaging system (Sequoia 512, Siemens, Mountain View, CA, USA) at low mechanical index (0.2).

Tumor volume was serially measured up to Day 15 using high-resolution 3-D ultrasound (Vevo 2100, VisualSonics, Toronto, Canada), after which the mice were euthanized using anesthesia (5% of Isoflurane for 5 min) [23]. Data for tumor volume vs. time were fit to the exponential function *V(t)* = *V*_0_*e^kt^*, where *V*_0_ represents the initial tumor volume and *t* represents time in days. Anti-tumor efficacy was evaluated by comparing normalized tumor volumes, tumor doubling time calculated as *DT* = (ln 2)/*k*, and survival rate.

#### Histologic analysis

Tumors were harvested post-mortem, fixed in 10% formalin, embedded in paraffin, and stained with Hematoxylin and Eosin (H&E). TUNEL assays were performed on tumor sections using an *In situ* Apoptosis Detection kit (ab206386, Abcam Inc., Cambridge, MA, USA) as per the manufacturer’s protocol. A similar process was followed for Ki67 analysis of proliferation using Ki67 primary antibody (ab15580) [26].

#### Real time quantitative PCR

The tumor tissues in TRIzol reagent (ThermoFischer Scientific, Waltham, MA, USA) were homogenized using an Ultrasonic Processor (Heat Systems, XL-2020). Chloroform (200 μL) was added to 1 mL of TRIzol solution and centrifuged at 12,000 g for 15 min at 4°C, and 200 μL of aqueous upper layer was pipetted into an RNAse-free centrifuge tube. After subsequent treatment with isopropyl alcohol and 75% ethanol, the RNA pellet was dissolved in DEPC water. Complementary DNA was prepared from 2 μg of total RNA using TaqMan reverse transcription kit (Applied Biosystems, Foster City, CA, USA). RT-PCR amplifications were performed with the SYBR™ Green PCR Master Mix (Thermo Scientific, Waltham, MA, USA) using an Applied Biosystems 7900HT instrument (Carlsbad, CA, USA). Gene expression levels were quantified using the ΔΔCt method. GAPDH RNA levels were utilized to normalize the relative gene expression levels (see primary sequence details in the S1 File).

### Statistical analysis

The data are presented as mean ± standard deviation. Analysis of variance (ANOVA) was performed to determine if there were differences among the experimental groups. If a difference was found (ANOVA *p*-value <0.05), *post hoc* Student’s t-testing was performed to determine wherein the differences resided, where significant differences between means was defined as *p*<0.05.

## Results

### Characterization of STAT3 decoy-loaded microbubbles

The STAT3-MB had an average diameter of 2.45±0.35 μm with an average concentration of 2×10^9^ microbubbles/mL and a loading capacity of 10 μg of STAT3 decoy per 1×10^9^ microbubbles. The STAT3-LPX had an average diameter of 3.50±0.29 μm with an average concentration of 2×10^9^ microbubbles/mL and a loading capacity of 50 μg of STAT3 decoy per 1×10^9^ microbubbles (5-fold higher than STAT3-MB).

### Effect of UTMC-mediated STAT3 decoy delivery on CAL33 cell viability *in vitro*

**Fig 1:**
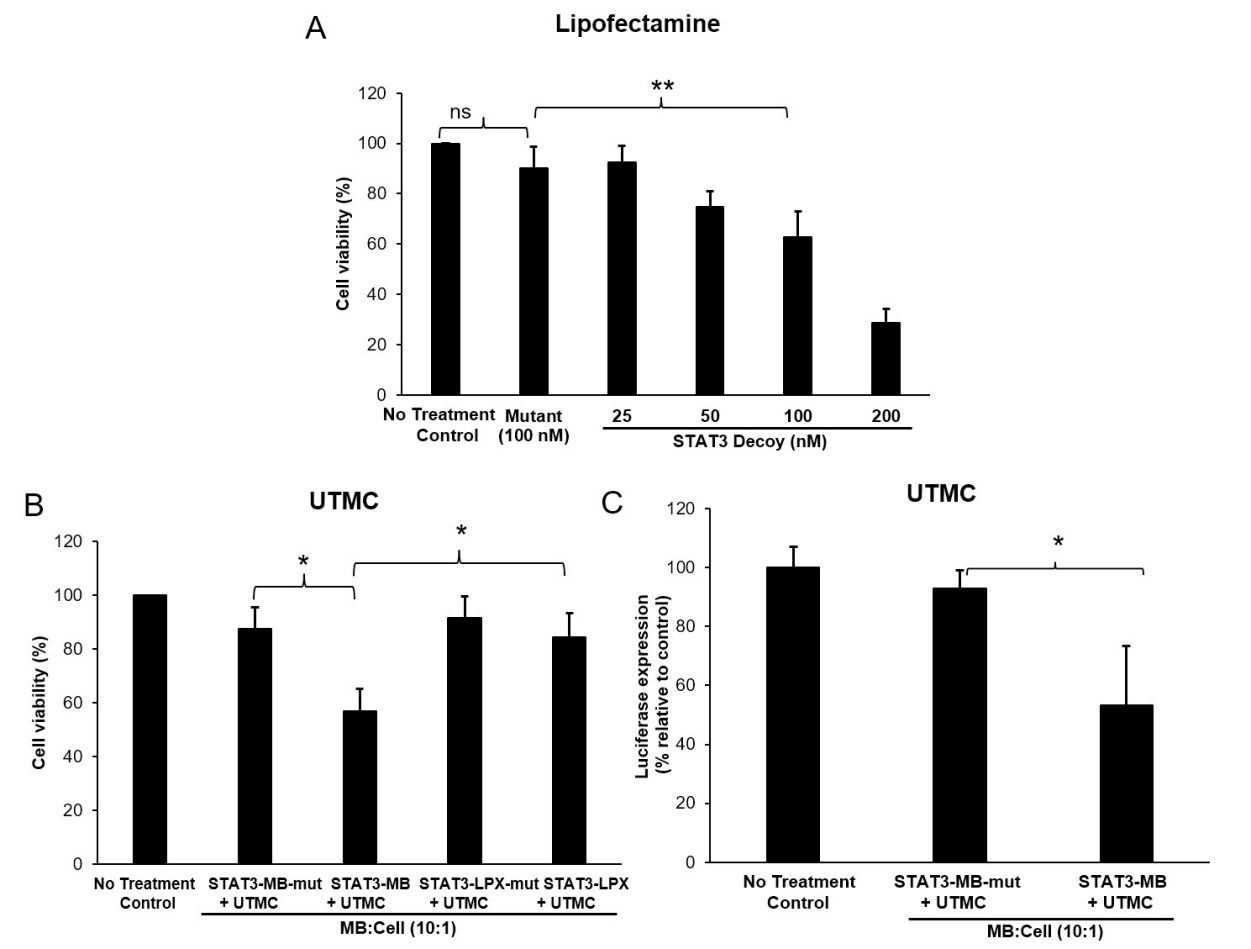
Anticancer activity of STAT3 decoy *in vitro*. (A) Cell viability analysis (MTT) of CAL33 cells upon treatment with various concentrations of STAT3 decoy complexed with lipofectamine. Cell viability decreased with an increase in STAT3 decoy concentration (**p<0.001). Data are presented as mean ± SD (*n* = 6 per group). (B) Cell viability analysis (MTT) after UTMC treatment with STAT3 decoy loaded microbubbles. STAT3-MB + UTMC caused significantly greater reduction in cell viability compared to that of mutant control. STAT3-MB + UTMC also showed greater cell killing capability compared with STAT3-LPX + UTMC (*p<0.05). Data are presented as mean ± SD (*n* = 6 each group). (C) Luciferase expression in cultured CAL33 cells expressing luciferase driven by a STAT3-responsive promoter after treatment with STAT3-MB + UTMC or STAT3-MB-mut + UTMC. STAT3-MB + UTMC caused significant knockdown of Luciferase expression compared with STAT3-MB-mut + UTMC (*n* = 3 each group, *p<0.05).

The activity of STAT3 decoy delivered via the various formulations against CAL33 cells was evaluated *in vitro* using MTT viability assays and apoptosis assays. STAT3 decoy delivered via lipofection induced a dose-dependent cytotoxic effect in CAL33 cells (Fig 1A). STAT3 decoy had a greater cytotoxic effect than its mutant control when delivered by lipofectamine (*p*<0.001), and the cell viability of no treatment control and mutant treated cells was similar. These lipofectamine data confirm that the STAT3 decoy was cytotoxic and the mutant decoy was not.

The *in vitro* cytotoxicity of STAT3-MB + UTMC vs. STAT3-LPX + UTMC was compared using MTT assay. STAT3-MB + UTMC resulted in significantly lower CAL33 cell viability (56.8±8.4%) compared to that of STAT3-LPX + UTMC (84.5±8.8%, *p*<0.05) (Fig 1B). Based on these data, the STAT3-MB formulation was used for the remaining *in vitro* and *in vivo* experiments.

In CAL33 luc^+^ cells where luciferase expression is driven by a STAT3-responsive promoter, STAT3-MB + UTMC caused a ~45% reduction in luciferase activity which was significantly (*p*<0.05) greater than that of mutant microbubble (<10%) (Fig 1C). On the target gene protein expression level, STAT3-MB + UTMC-treated tumors exhibited a strong trend towards decreased expression of Bcl-xL (normalized by GAPDH) (36.9±25.7%) compared to the mutant control (94.5±50.1%, *p*<0.15).

**Fig 2:**
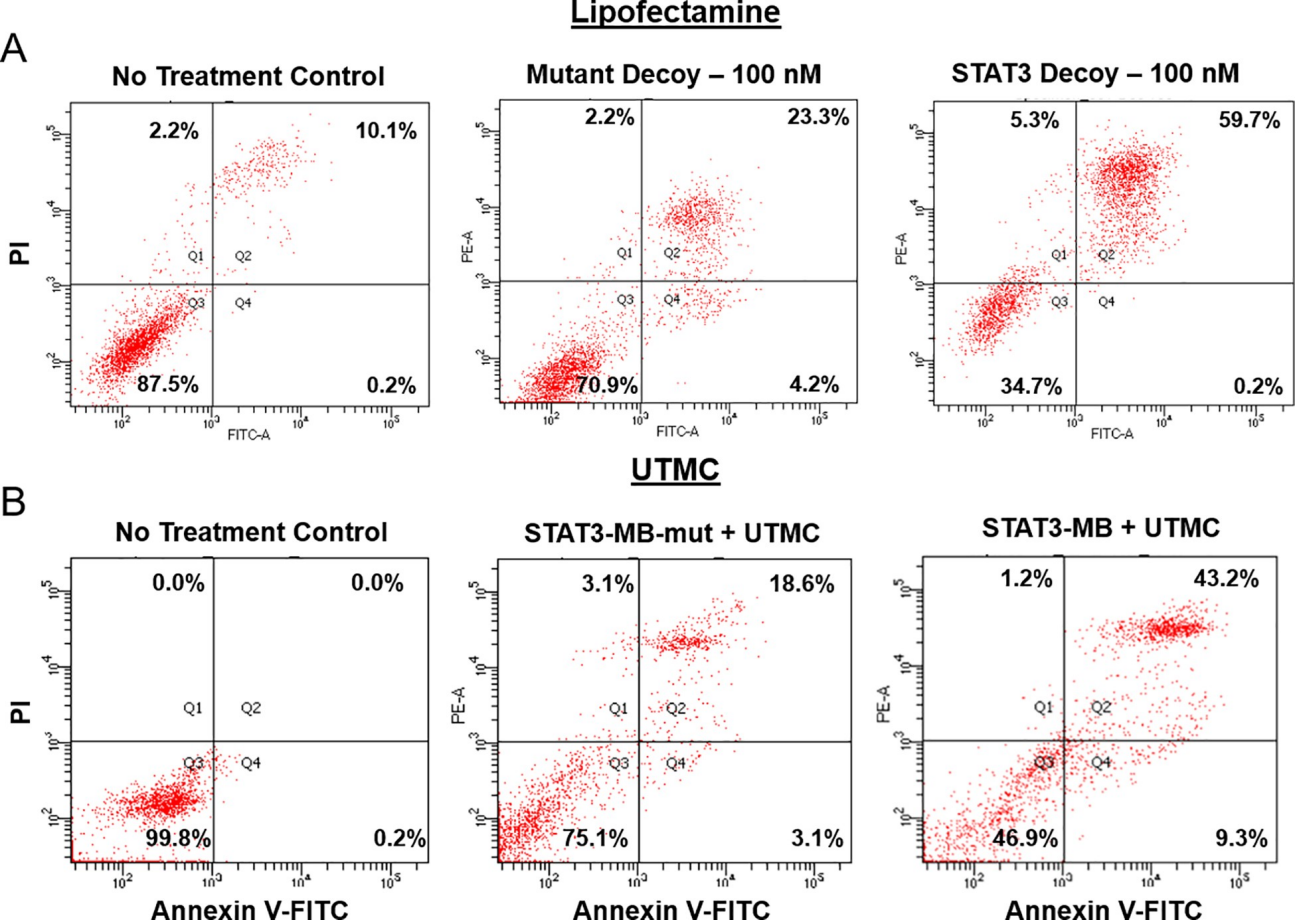
Flow cytometric analysis of apoptosis in CAL33 cells using Annexin V-FITC and propidium iodine (PI) staining. (A) There was a higher proportion of apoptosis among cells treated with STAT3 decoy complexed to lipofectamine compared to untreated or mutant decoy-treated cells. (B) There was a higher proportion of apoptosis among cells treated with STAT3-MB + UTMC compared to untreated cells or cells treated with STAT3-MB-mut + UTMC. Lower Left (Q3)–viable cells; Upper Left (Q1)–necrosis; Lower Right (Q4)–early apoptosis; Upper Right (Q2)–late apoptosis.

Fig 2 shows the flow cytometry data interrogating cells for the presence of apoptosis in response to lipofectamine or UTMC treatments, where early apoptosis (lower right quadrant) is characterized by Annexin V (+) and PI (-) staining, and late apoptosis (upper right quadrant) is characterized by Annexin V (+) and PI (+) staining. Fig 2A shows data from CAL33 cells receiving no treatment (left), lipofectamine-mediated delivery of 100 nM mutant decoy (middle), or STAT3 decoy (right), indicating STAT3-mediated induction of apoptosis. Fig 2B depicts data from experiments with CAL33 cells receiving no treatment (left), UTMC-mediated delivery of mutant decoy-microbubble (middle), or STAT3-MB (right). Compared to untreated cells or STAT3-MB-mut + UTMC treated cells, STAT3-MB + UTMC treatment was associated with reduced viability and increased apoptosis.

### UTMC-mediated STAT3 decoy delivery inhibits CAL33 tumor growth *in vivo*

**Fig 3:**
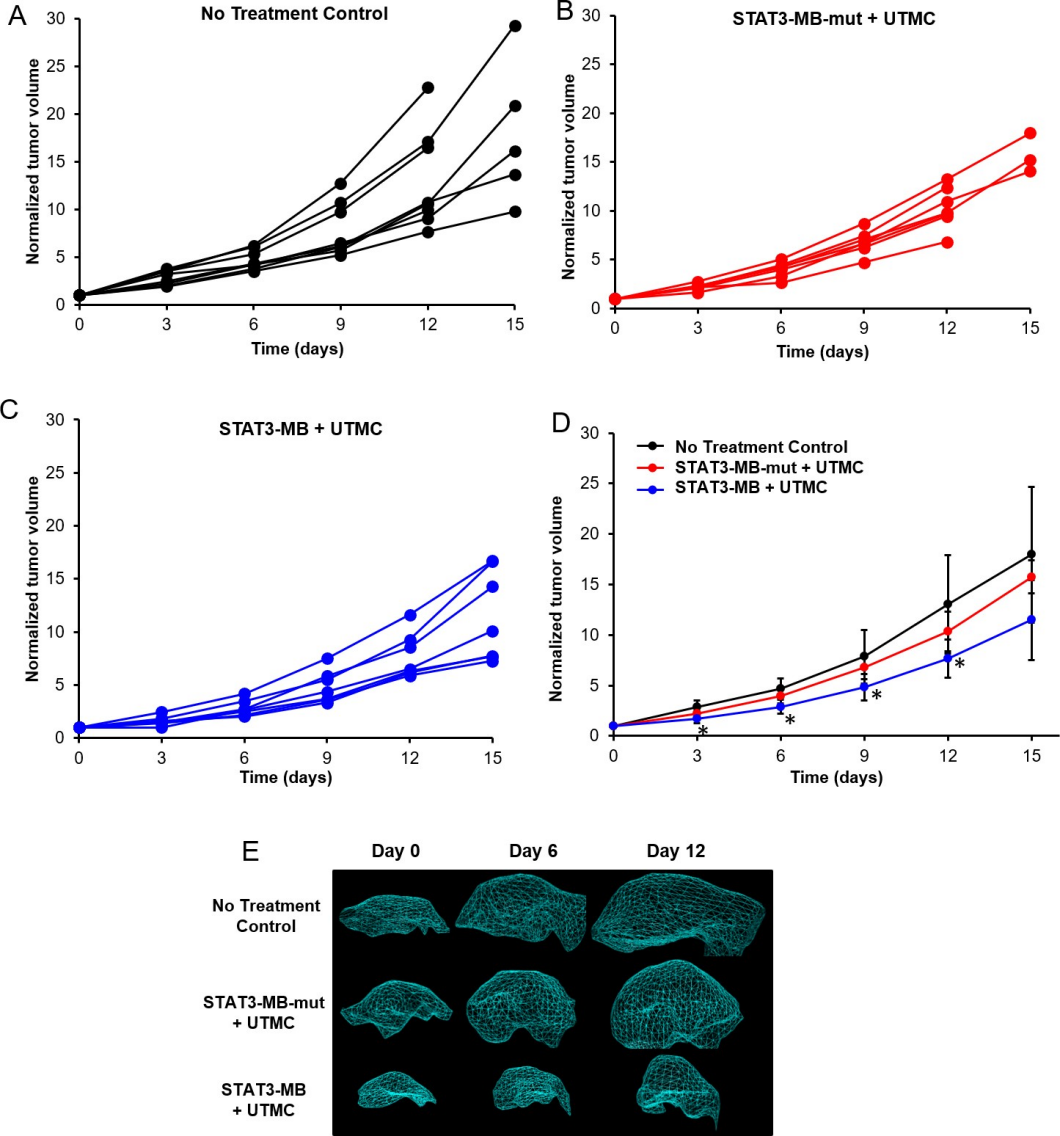
*In vivo* efficacy of UTMC-mediated STAT3 decoy delivery against human HNSCC xenografts. Tumor volumes (normalized to initial volume on day 0) of CAL33 tumor-bearing mice that received (A) no treatment control (*n* = 8), (B) STAT3-MB-mut + UTMC (*n* = 7), and (C) STAT3-MB + UTMC (*n* = 7). (D) Mean normalized tumor volumes of CAL33 tumor-bearing mice for each of the treatment groups. The normalized volumes for STAT3-MB + UTMC group were significantly lower than STAT3-MB-mut + UTMC and no treatment control on days 3, 6, 9, and 12 (*p<0.05). (E) Representative 3-D reconstruction of tumor volumes.

**Fig 4:**
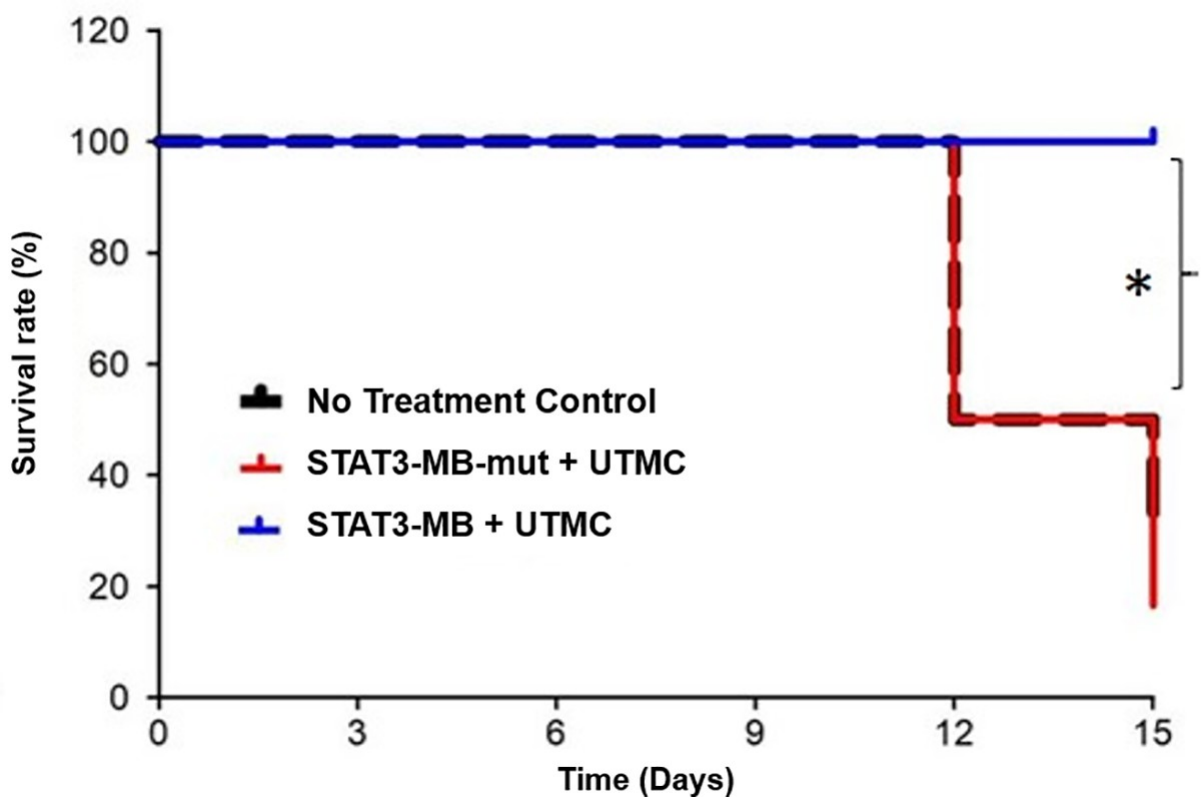
Kaplan–Meier survival log-rank analysis of the CAL33 tumor-bearing mice receiving different treatments. The survival for STAT3-MB + UTMC group was significantly longer compared with STAT3-MB-mut + UTMC and no treatment control (*p<0.05).

STAT3-MB + UTMC (*n* = 7) reduced tumor growth compared to that in untreated control (*n* = 8) and STAT3-MB-mut + UTMC-treated mice (*n* = 7) (Fig 3). Significant differences (*p*<0.05) in tumor volumes were observed between the STAT3-MB + UTMC group and the two control groups starting at Day 3 and continuing until the end of study period (Fig 3D). By Day 12, the STAT3 decoy treated mice had a 2-fold reduction (*p*<0.05) in tumor volume compared to that of the no treatment control group and a 1.5-fold reduction (*p*<0.05) compared to that of STAT3-MB-mut + UTMC, respectively (Fig 3D). Mice treated with STAT3-MB-mut + UTMC did not exhibit a significant difference in tumor volume compared to that of untreated control mice at any point during the study. The individual growth curves for each experimental group are shown in panels A through C in Fig 3. It should be noted that in the untreated control and mutant-treated control groups, several mice were euthanized according to protocol before Day 15 due to tumor growth exceeding 1000 mm^3^, thereby precluding tumor volume comparisons between treatment groups at this time point (Fig 3D). Fig 3E depicts representative serial tumor volumes derived from 3D reconstructed ultrasound images. Aligned with this finding, UTMC-mediated STAT3 decoy delivery increased tumor doubling time compared to that of untreated control (4.2±0.4 vs 3.5±0.4 days, *p*<0.05) and mutant control decoy (3.7±0.4, *p* = 0.056), resulting in reduced mortality of the STAT3 decoy treated mice. Kaplan-Meier curves (Fig 4) indicated that by Day 15 all mice in the STAT3-MB + UTMC treated group were still alive compared to only 33% and 14% for STAT3-MB-mut + UTMC and untreated groups, respectively (*p*<0.05).

### UTMC-mediated STAT3 decoy delivery downregulates STAT3 target genes in CAL33 tumors *in vivo*

**Fig 5:**
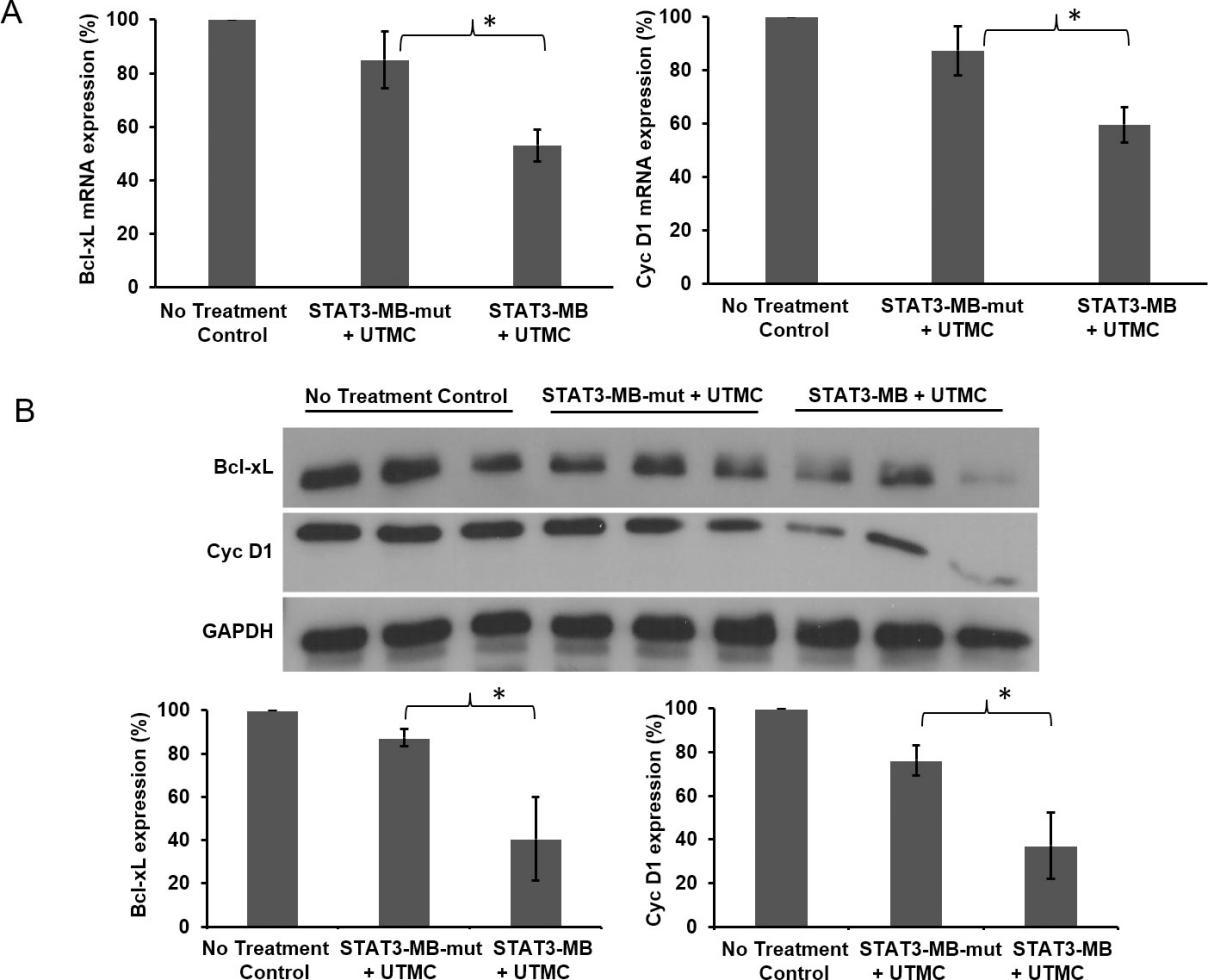
Target gene expression in CAL33 tumors 48 hours after STAT3-MB + UTMC. (A) RT-PCR and (B) Western blot analysis of gene expression in CAL33 tumor samples. Tumors excised from STAT3-MB + UTMC group (*n* = 3) showed significant (*p<0.05) downregulation of target genes Bcl-xL and cyclin D1 (Cyc D1), compared to that of tumors excised from no treatment control (*n* = 3) and mutant-treated (STAT3-MB-mut + UTMC) groups (*n* = 3).

UTMC-mediated delivery of STAT3 decoy or mutant decoy was performed in a separate group of CAL33 tumor-bearing mice, and tumors were harvested 48 h later and analyzed for target gene expression. Compared to mutant decoy, STAT3 decoy caused a significant decrease in mRNA and protein expression of target genes Bcl-xL and cyclin D1 (*p*<0.05) (Fig 5). UTMC-mediated delivery of mutant control decoy did not confer significant decreases in the respective protein expression compared to that of no treatment control.

### Histologic analysis of tumor tissues

**Fig 6:**
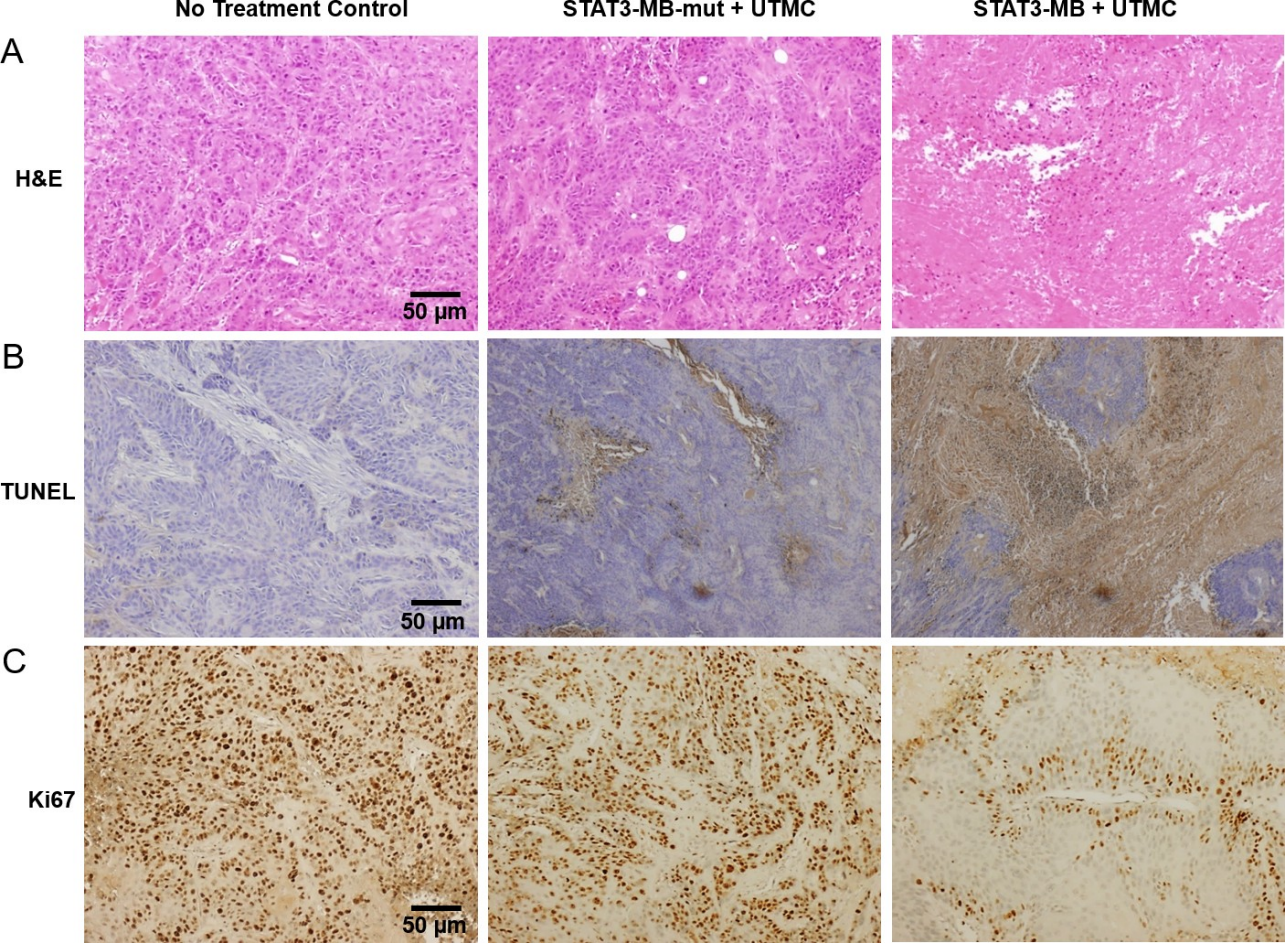
Immunohistochemical analysis of excised tumor tissues. Representative images of (A) Hematoxylin and Eosin (H&E)-, (B) *in situ* cell apoptosis (TUNEL)-, and (C) immunological Ki67-stained sections of excised CAL33 tumors. There were more TUNEL positive (apoptotic) cells and fewer Ki-67 (proliferating) cells in tumors treated with STAT3-MB + UTMC compared to untreated and mutant-treated tumors.

On H&E staining, STAT3 decoy treated tumors showed fragmentation of cells and apoptotic condensations (Fig 6A). STAT3-MB treated tumors demonstrated extensive regions of apoptotic (TUNEL-positive) cells compared to no treatment control and mutant-treated tumors (Fig 6B). STAT3 decoy-treated tumors showed markedly less Ki-67 staining compared to other groups, indicating greater inhibition of tumor cell proliferation (Fig 6C).

## Discussion

The main finding of this study is that lipid microbubbles loaded with a cyclic STAT3 decoy oligonucleotide, in the presence of image-guided therapeutic ultrasound, inhibit growth of human HNSCC tumors and suppress downstream target gene expression. As the first demonstration of UTMC-mediated delivery of STAT3 decoy to cancer cells of human origin, these data set the stage for clinical translation of ultrasound-mediated delivery of STAT3 decoy for the treatment of patients with head and neck tumors.

STAT3 is constitutively hyperactivated in many cancers, including head and neck cancers. Multiple signaling pathways converge at STAT3, making it a master regulator mediating molecular events that result in cancer progression. The STAT3 decoy oligonucleotide binds to activated STAT3 with a high specificity and inhibits STAT3-mediated gene transcription and cancer cell proliferation [27]. It has been shown that intratumoral injection of STAT3 decoy downregulates the expression of STAT3 target genes in humans [12]. Intratumoral injection, however, is not clinically practical. Other studies using intravenous injections of STAT3 decoy [14, 24] utilized daily, relatively high doses of decoy to effect tumor growth inhibition, highlighting that, as with all nucleic-acid therapeutics, systemic administration is an inefficient strategy for concentrating the therapeutic into tumor tissue and there is a need for more efficient targeted delivery platforms.

To this end, we developed an image-guided targeted delivery strategy employing oligonucleotide-loaded microbubbles that can be ultrasonically induced to deliver their payload exclusively to tumor as they transit through tumor microcirculation [28–31]. We previously demonstrated that UTMC treatment with STAT3 decoy-loaded cationic lipid microbubbles inhibited tumor growth in mice harboring murine skin squamous cell (SCC-VII) tumors [23]. To take the next clinical translational step, the goal of the present study was to test the hypothesis that UTMC-mediated delivery of STAT3 decoy inhibits growth of human head and neck squamous cell carcinoma *in vitro* and *in vivo*. Further, we sought to determine whether increasing STAT3 loading on the microbubbles above that previously described by us [23] would increase tumor inhibition beyond that achieved by our prior formulation. Accordingly, we employed *in vitro* studies using cultured human head and neck carcinoma cells (CAL33) and *in vivo* experiments using nude mice bearing CAL33 xenograft tumors.

Our prior cationic lipid microbubble formulation (STAT3-MB) maximally loads 10 μg of STAT3 decoy on 1×10^9^ microbubbles/mL [23]. To increase the STAT3 decoy dose in the carrier, we designed a STAT3-loaded liposome microbubble construct (STAT3-LPX). Multivalent cationic lipids increase the number of charges on unit surface areas, thereby enhancing nucleic acid binding. Here we demonstrated that the cationic liposome-conjugated microbubbles were indeed capable of carrying 5 times the amount of decoy on the plain lipid microbubble. Interestingly, despite the higher loading capacity of STAT3-LPX, this formulation was less cytotoxic to CAL33 cells *in vitro*. We interpret this to indicate that increasing the STAT3 dose via liposomal incorporation on to the microbubble is not equivalent to higher effective bioavailability of the decoy. It is known that release of payloads from liposomal carriers may be inefficient or slow [32, 33], and we have previously shown *in vitro* that release of doxorubicin from liposome-microbubble constructs is increased by long tone burst ultrasound [34]. In the present study, we employed shorter tone bursts; therefore, it is possible that the STAT3 decoy was not sufficiently released from the liposome with our pulsing scheme, potentially causing inferior cytotoxicity of STAT3-LPX compared with STAT3-MB, despite higher STAT3 loading on the microbubble construct. Nonetheless, based on the *in vitro* data, we selected the STAT3-MB formulation for the remaining experiments.

We demonstrated that compared to negative controls (no treatment control or STAT3-MB-mut + UTMC), cultured (human) CAL33 cells treated with STAT3-MB + UTMC experienced: (1) greater cytotoxicity as shown by MTT assay; (2) more apoptosis as shown by flow cytometry; and (3) a strong trend in decreased protein expression of downstream target genes Bcl-xL as shown by Western blot. Aligned with the *in vitro* results, we found that nude mice bearing CAL33 tumors experienced less tumor growth and longer survival when treated with STAT3-MB + UTMC compared to the negative control groups, and this was associated with downregulation of target gene Bcl-xL and Cyc-D1 expression at the RNA transcript and protein levels. The tumor immunohistochemistry data indicated more apoptosis and less proliferation activity in the STAT3-MB + UTMC treated tumors. Importantly, UTMC-mediated delivery of mutant control decoy did not result in tumor growth suppression or target gene downregulation compared to untreated control mice, indicating that the therapeutic effect was not simply due to the delivery of ultrasound.

The mechanisms for UTMC-induced enhancement of nucleic acid therapeutics are incompletely understood. UTMC capitalizes on the unique acoustic behavior of gas-filled microbubbles in an ultrasound field, whereby the microbubbles vibrate, or cavitate, in response to the mechanical perturbations of ultrasound waves. We and others [29, 30, 35–37] have shown that microbubble cavitation induced by ultrasound at the transmit frequencies and acoustic pressures used in diagnostic imaging and in the current study causes shear stress, leading to the formation of pores or holes spanning the apical and basal cell membranes, and which spontaneously heal within minutes (“sonoporation”). We have also shown that sonoporation allows cytoplasmic entry of cell-impermeant molecules, suggesting that therapeutic oligonucleotides can enter directly into the cell and avoid degradation by the endocytosis pathway, which typically limits bioavailability of oligonucleotides delivered using other platforms. Furthermore, we have also shown *in vitro* that after a sonoporation event, junctions between confluent endothelial cells form and remain open for hours [30], and others have shown that UTMC can permeabilize the blood brain barrier [38], suggesting that transient endothelial hyperpermeability may be another mechanism by which UTMC facilitates delivery of oligonucleotides beyond the vascular endothelial barrier. It should be noted that while most cells remain viable after an ultrasound pulse specifically configured for sonoporation, some cells are unable to reseal their cell membrane and die, particularly when the pore is large [37]. Indeed, prior studies have tested multiple acoustic regimens to minimize cytotoxicity related to UTMC alone. The potential for inherent toxicity from the UTMC itself may account for why tumors treated with the mutant decoy-loaded microbubble + UTMC experience a mild reduction, albeit statistically insignificant, in tumor growth.

Our study demonstrated significant therapeutic efficacy of our UTMC platform compared to that of other reports using different STAT3 decoy delivery platforms and higher STAT3 decoy doses in HNSCC models. We previously reported tumor regression and inhibition of STAT3 signaling in HNSCC xenografts after intravenous administration of STAT3 decoy (5 mg/kg/day), 5 days per week for 2 weeks [13, 14]. We recently found tumor growth inhibition of 201T- and H1975-derived xenografts after the daily intravenous administration of STAT3 decoy (5 mg/kg/day), 5 days per week for 14–20 days [24]. These studies used a 5 mg/kg dose of STAT3 decoy, whereas we demonstrated a similar therapeutic efficacy at a much lower dose of <0.5 mg/kg for each of only 3 UTMC treatments. Although it remains to be proven, such data suggest that UTMC confers a comparable intratumoral concentration of decoy to what is achieved by an order of magnitude greater dose of intravenously administered STAT3 decoy. Conceivably, the endocytosis-independent drug entry and transient endothelial barrier hyperpermeability induced by UTMC (as suggested by the *in vitro* studies described above), combined with the release of the decoy specifically as the loaded microbubbles transit the tumor, concentrates delivery of the decoy within the insonified tumor. The order of magnitude lower dose requirement conferred by UTMC may have clinical translational benefit by reducing the overall dose required to achieve therapeutic efficacy while reducing off-target effects.

Some limitations to our study bear mention. Despite the fact that UTMC-based STAT3 decoy delivery inhibited tumor growth, complete regression of tumor was not achieved and tumor growth recommenced after the treatment was terminated (Day 7). The number of UTMC treatments we could give was inherently limited because frequent general anesthesia, as would be necessary for daily microbubble intravenous infusion and UTMC in these mice, would not be well tolerated. In future clinical applications, daily peripheral intravenous microbubble administration with UTMC is highly feasible, and the opportunity to more aggressively promote tumor regression would be afforded.

The liposome microbubble conjugated formulation (STAT3-LPX) carried 5-fold greater STAT3 decoy than the cationic lipid formulation (STAT3-MB) yet was not more effective *in vitro*. Although we did not specifically test for dissociation of the decoy from the liposome in the serum-containing culture media used in our *in vitro* studies, previous reports have shown excellent binding and stability of nucleic acids in liposomes in a serum environment up to 50% [39–42], while the serum concentration of our culture media was only 2%. It is thus unlikely that “leakiness” of the liposome accounted for the decreased efficacy of STAT3-LPX formulation *in vitro*. Nonetheless the inferior performance of the STAT3-LPX formulation does not negate the concept that formulations with higher MB loading doses could achieve higher UTMC therapeutic efficacy. Rather, such data indicate that other factors, including the acoustic regimen and its interaction with the specific microbubble formulations, should be studied to optimize the bioavailability of decoy delivered using new, more highly loaded, microbubble species.

## Conclusion

To the best of our knowledge, this is the first study to demonstrate the efficacy of UTMC-based STAT3 decoy in human cancer cells. Our study demonstrated the ability of STAT3 decoy microbubbles and ultrasound to inhibit the growth of human HNSCC and downregulate target genes involved in signaling pathways that contribute to tumor cell survival and proliferation. Significant therapeutic effects were observed at a fraction of the STAT3 decoy dose required to achieve the same inhibitory effect from intravenously injected decoy. Taken together, our study lays important groundwork for the clinical translation of UTMC for targeted delivery of STAT3 decoy, and possibly other therapeutic oligonucleotides, for the treatment of HNSCC and other solid tumors.

## Supporting information

S1 File (DOCX)

## Abbreviations list

STAT3: signal transducer and activator of transcription 3
STAT3-MB: STAT3 decoy-loaded microbubbles
STAT3-LPX: STAT3 decoy-loaded liposome-conjugated microbubble complexes
UTMC: ultrasound-targeted microbubble cavitation
MB: microbubble
ON: oligonucleotide
HNSCC: head and neck squamous cell carcinoma
