# Supplementary material for: STAT3 decoy oligonucleotide-carrying microbubbles with pulsed ultrasound for enhanced therapeutic effect in head and neck tumors
Source: PLoS One. 2020 Nov 18;15(11):e0242264. doi: 10.1371/journal.pone.0242264 (PMC7673576; doi:10.1371/journal.pone.0242264)
Supplement: S1 File — (DOCX) [file pone.0242264.s001.docx]

**STAT3 decoy oligonucleotide-carrying microbubbles with pulsed ultrasound for enhanced therapeutic effect in head and neck tumors**

**Short title:** STAT3 decoy in head and neck tumors

Thiruganesh Ramasamy^1^, Xucai Chen^1^, Bin Qin^1^, Daniel E. Johnson^2^, Jennifer R. Grandis^2^, Flordeliza S. Villanueva^1,*^

^1^ Center for Ultrasound Molecular Imaging and Therapeutics, Pittsburgh Heart, Lung, Blood and Vascular Medicine Institute, University of Pittsburgh, Pittsburgh, PA, USA

^2^ Department of Otolaryngology-Head and Neck Surgery, University of California San Francisco, San Francisco, CA, USA

^*^ **Corresponding author**

**E-mail:** [villanuevafs@upmc.edu](mailto:villanuevafs@upmc.edu) (FSV)

**ONLINE SUPPLEMENTAL MATERIAL**

**Detailed Methods**

**Apoptosis assay *in vitro***

A quantitative apoptosis assay was flow cytometrically performed using Annexin V-FITC and propidium iodine (PI) staining (BioLegend Kit, San Diego, CA, USA) of the CAL33 cells. Briefly, CAL33 cells were seeded (seeding density 1×10^5^ per well) in a 12-well plate and allowed to grow for 24 h. The cells were then treated with 25-200 nM molar concentration of STAT3 decoy (lipofectamine complexes), STAT3-MB + UTMC, or STAT3-LPX + UTMC, incubated for 48 h, then extracted, washed twice with cold PBS, and centrifuged. The cell pellet was re-dispersed with 100 µL of binding buffer and stained with 2.5 µL of Annexin V-FITC and 5 µL of PI and incubated for 15 min under dark conditions. The volume was made up to 1000 µL and cell samples were analyzed using a FACS Verse Flow Cytometer (BD Biosciences, San Jose, CA, USA). A cell count of 10,000 was acquired for each sample.

Apoptosis was further studied using Hoechst 33342 staining in separate samples. The cells were seeded and treated with STAT3 decoy and lipofectamine or STAT3-MB + UTMC, using protocols as described above. The cells were stained with Hoechst 33342 (10 µg/mL), incubated for 15 min, fixed with 4% paraformaldehyde, then washed twice and observed under an inverted fluorescence microscope (IX81, Olympus America, Central Valley, PA, USA).

**Western Blot analysis**

Target gene expression changes resulting from the various STAT3 decoy delivery strategies were evaluated using Western Blot. CAL33 cells treated with UTMC were incubated for 48 h, washed twice with PBS, detached in the presence of scrapping buffer, centrifuged, and lysed using lysis buffer. The extracted proteins were separated using SDS-PAGE gel and transferred to PVDF membrane (Millipore, MA, USA). The membrane was blocked with 5% skim milk in TBS with 1% Tween 20 (TBST). The membrane was incubated with Bcl-xL, cyclin D1, and GAPDH antibody (diluted in 1:1000 of 2% BSA in TBST as per the manufacturer’s recommendation) overnight at 4°C under gentle shaking. The membrane was washed thrice with TBST and incubated with respective secondary antibody (2:10,000) for 1 h and then washed again with TBST. The membrane was exposed with HRP substrate (Luminata^TM^ Classico Western HRP Substrate, Billerica, MA, USA) and bands were developed using an imaging system (AFP Imaging Corp, USA). For *in vivo* studies, harvested tumor tissue was homogenized in buffer containing 10 mM Tris, 5 mM EDTA, 50 mM NaCl, 30 mM Na_4_P_2_O_7_, 1 mM Na_3_VO_4_, 1% triton X-100, pH 7.6 supplemented with 1 Complete Mini Tablet (Roche Applied Science, Penzberg, Germany) used to make 10 mL. The protein lysates were used to perform Western blot analysis as described above.

**Real time quantitative PCR**

The sequences for primers were as follows:

mBcl-xL Forward: AGCAGGTAGTGAATGAACTCTTTCG

mBcl-xL Reverse: CCATCCAACTTGCAATCCGACTC

mCyclin-D1 Forward: ATGGAACACCAGCTCCTGTGCTGCG,

mCyclin-D1 Reverse: TCAGATGTCCACATCTCGGACGTCG

mGAPDH Forward: TGCAGTGGCAAAGTGGAGATT

mGAPDH Reverse: TGCCGTTGAA TTTGCCGT


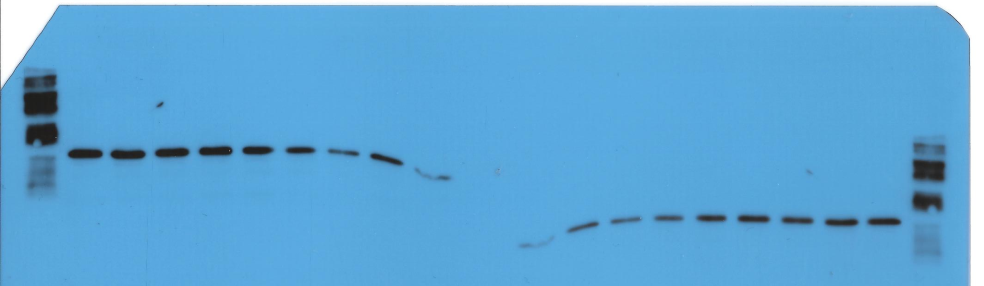


Original blots of Cyclin D1.


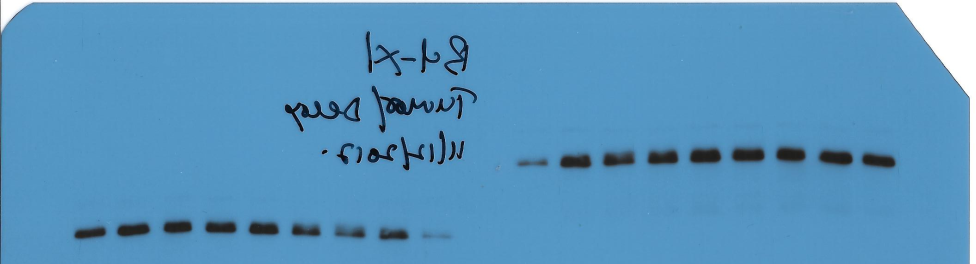


Original blots of Bcl-XL.


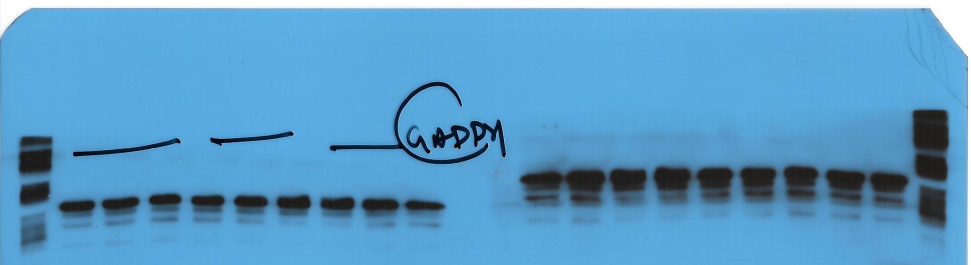


Original blots of GAPDH.
